# Assessment of Welfare in Zoo Animals: Towards Optimum Quality of Life

**DOI:** 10.3390/ani8070110

**Published:** 2018-07-04

**Authors:** Sarah Wolfensohn, Justine Shotton, Hannah Bowley, Siân Davies, Sarah Thompson, William S. M. Justice

**Affiliations:** 1 School of Veterinary Medicine, University of Surrey, Guildford, Surrey GU2 7AL, UK; hb00172@surrey.ac.uk (H.B.); sd00257@surrey.ac.uk (S.D.); st00445@surrey.ac.uk (S.T.); 2 Marwell Wildlife, Colden Common, Winchester, Hampshire SO21 1JH, UK; justines@marwell.org.uk (J.S.); willj@marwell.org.uk (W.S.M.J.)

**Keywords:** zoo animals, welfare, quality of life, lifetime experience, animal welfare assessment grid

## Abstract

**Simple Summary:**

Maintaining a high standard of animal welfare is essential in zoos, and methods of animal welfare assessment should aim to evaluate positive as well as negative states. The indicators that are useful in assessing these are discussed as there is huge variability in the available information about the natural biology for some zoo species. Wild baselines are not always the most accurate indicator of what is right for an animal in captivity, which makes the identification of factors to include within species-specific welfare assessment even more challenging. There is no “one size fits all” welfare strategy as it should account for the range of biological requirements and needs, which it is not possible to define for some zoo species. The different approaches for welfare assessment are reviewed, including the development of the Animal Welfare Assessment Grid which offers an evidence-based tool for continual welfare assessment, using technology where appropriate, to facilitate decision making and lead to improvements in the animals’ quality of life.

**Abstract:**

Zoos are required to maintain a high standard of animal welfare, and this can be assessed using a combination of resource-based and animal-based indices usually divided into behavioural indicators, physiological indicators and clinical/pathological signs. Modern animal welfare assessments should aim to encompass positive affective states and the indicators that are useful in assessing these are discussed. When developing factors to be scored for each species, there is huge variability in the available information about the natural biology for some zoo species and even less information concerning those animals in captivity. Wild baselines are not always the most accurate indicator of what is right for an animal in captivity, which makes the identification of factors to include within species-specific welfare assessment even more challenging. When planning a welfare strategy for any species, it is important that the full range of their biological requirements and needs are considered, but this can be challenging for some zoo species and it is not possible to define a “one size fits all” welfare strategy. The different approaches for welfare assessment are reviewed, including the development of the Animal Welfare Assessment Grid which offers an evidence-based tool for continual welfare assessment, using technology where appropriate, to facilitate decision making and lead to improvements in the animals’ quality of life.

## 1. Introduction

Ensuring a high standard of zoo animal welfare is important for both ethical and legislative reasons [1]. In the UK, there are several pieces of legislation governing the welfare of animals in zoos. These include the Zoo Licensing Act 1981 (ZLA), EC Council Directive 1999/22/EC and the Animal Welfare Act (England and Wales) 2006. The ZLA requires animals to be accommodated under conditions that satisfy the biological and conservation requirements of the species. This includes an environment well adapted to meet the physical, psychological and social needs of the species and a high standard of husbandry with a developed programme of preventative and curative veterinary care [2]. This legislation has been further augmented by the Secretary of State’s Standards of Modern Zoo Practice which states the expected standard of animal welfare in zoos and is periodically updated to reflect recent advances in understanding [3]. More detailed methods of assessment are laid out in the Zoos Expert Committee’s handbook (2012) [4]. The handbook suggests using a combination of resource-based and animal-based indices, and that opportunities are provided for animals to experience positive emotional states rather than just avoiding negative states. Assessment methods are divided into three groups: behavioural indicators, physiological indicators and clinical/pathological signs. It suggests best practice is to carry out welfare audits once or twice yearly [4].

When planning a welfare strategy of any species, it is important that the full range of their biological requirements and needs are considered. Anything known about an animal’s natural history is an important resource for understanding its requirements and the environmental parameters under which it should be kept in captivity. Therefore, published research articles on the species’ biology may assist in the construction of a welfare strategy in the absence of specific welfare provision guidance. However, the current state of knowledge for many species is not comprehensive and so it is not always possible to use this as a gold standard against which to assess animal welfare [5], and it is not possible to define a “one size fits all” welfare strategy [6].

The concept of a life worth living or even a good life [7] introduces the idea of considering the temporal component of welfare across the duration of the animal’s life and the need to assess quality of life. Frequently welfare assessment simply looks at the presence of pain and the incidence and severity of disease or injury, reflecting current health status and not considering the total components of welfare. For zoo animals, this is particularly relevant since the majority of zoo animals are managed in a way that aims to ensure reasonably good health, but there is sometimes insufficient emphasis on overall welfare including the adequacy of the environment and the opportunity to express a range of normal behaviours. However, these may be contrary to the desire to show off the animal and provide a close up experience with animals to a revenue generating visiting public. Additionally, for zoo animals that are destined to be returned to the wild or are to be used in international breeding programmes, while their health may be optimal, there are plenty of other issues which can impinge negatively on their welfare. While travelling internationally to participate in breeding programmes may sound positive from a human perspective and for conservation reasons, for an animal, this will involve transportation, change in social hierarchy, change in personnel delivering care, a new environment and numerous stressors which could negatively impact on its welfare and quality of life.

The zoo industry has produced a number of guidance documents regarding animal welfare. The World Association of Zoos and Aquaria has recently produced an animal welfare strategy [8]. This document outlines the conduct expected from WAZA members, including animal welfare measures. Welfare has been defined according to the five freedoms (Freedom from hunger and thirst; Freedom from discomfort; Freedom from pain, injury and disease; Freedom to express normal behaviour; and Freedom from fear and distress) [9]. WAZA recommends that zoos and aquariums should apply the Five Domains welfare model (Nutrition, Environment, Health, Behaviour and Mental state [10]) to assess animal welfare by evaluating the four physical/functional domains and then considering the positive and negative affects generated by these factors within the Mental State domain [11].

## 2. Assessment of Welfare

The use of objective measurements of welfare to assess it quantitatively, rather than simply qualitatively, will assist with improving an animal’s quality of life. Evaluating the welfare impact of responses to any clinical interventions or changes in management; for example, transportation, or changes in housing need to be included. It is important not only to focus on the absence of suffering and abnormal behaviours, but also to aim to assess indicators of high welfare through assessing quality of life, positive affective states and “pleasure” [10,12,13,14,15,16,17,18,19,20]. Boissy et al. [21] reviewed the evidence supporting the existence of these positive affective states in animals and the explicit inclusion of positive welfare outcomes will allow for analyses which can yield more objective policies to improve welfare following retrospective reviews.

It has been suggested that improved welfare states can be achieved through positive anticipation, giving animals control of their environment, and rewarding them with a higher than anticipated reward—“positive contrast” [21,22]. Yeates and Main [13] reviewed positive welfare assessment and suggested that it should include both assessment of the resources available to the animal and the value it puts on them, together with the positive behavioural, psychological and physiological outcomes of using these resources and environments. However, they cautioned also to assess any long-term negative effects caused by the resources offered, as animals tend to choose what is rewarding in the short-term even where they have potential longer-term detrimental effects [13]. Modern animal welfare assessments should aim to encompass a measurement of these positive affective states as part of their assessment [23], and these measures should be incorporated also into zoo animal welfare assessments.

An animal’s response to a reduction in its welfare will be modified by a number of factors and so interpretation of the response will need to include consideration of the individual details of the animal, such as its species, age, and origin—which may all affect its response. The extent of any examination such as observation of feeding behaviour (quantity and pattern of feeding), physiological parameters (such as heart rate, respiration rate, body temperature, muscle tone, and colour of mucous membranes) and possibly biochemical indicators (such as enzyme levels) will depend on the species. The animal may appear dull, depressed, aggressive, or hyper-excitable, but particularly important are how such traits are at variance with its usual behaviour. Particular attention should be given to noticing any changes in gait, posture or facial expression. Vocalisation will also depend on the species and there are a wide variety of different noises produced by each species. The sound emitted may be outside the human auditory range and therefore go unnoticed but may be causing distress to others of the same species. These are very general descriptions on how to assess changes in welfare qualitatively, and the interpretation of such parameters will vary quite widely between observers, depending on their knowledge and experience of the species and the individual animal under observation.

In making an assessment, it is also necessary to consider whether the animal is juvenile or adult, any normal physiological variation (such as pregnancy) and the animal’s individual temperament. The subjective assessment of the welfare of animals may be based on anthropomorphic assumptions. For example, the assumption that situations or states which are distressing for humans will also be distressing for animals is not necessarily the case. This may lead to a tendency to overestimate any poor welfare experienced by animals in some situations, and underestimate it in others. Sometimes quite abnormal states may be classified by some observers as normal, simply because they occur commonly, for example, regurgitation may be seen frequently in non-human primates [24] but not necessarily recognised as abnormal by animal carers. It is much better to quantify the assessment being made so that it is objective, in order to be able to judge whether welfare has been improved, and to reduce inter-observer variability in making the assessment. When assessing animal welfare there should be assurances that this is not being done from an anthropogenic viewpoint but this can be helpful in the absence of an evidence base and will give the animal the benefit of the doubt. An awareness that certain environmental parameters which we fail to put emphasis on or provide for some species (such as temperature, access to water, etc.), may be valued by the animals much more highly than the factors we feel a duty to provide [17].

## 3. Physiological Assessment

There are various components that can be used to contribute to the objective assessment of an animal’s welfare. Traditional measures of welfare include indicators of physiological stress which focus on monitoring autonomic responses, such as changes in heart rate, respiration rate, blood pressure and/or temperature [25,26,27]. This requires either restraint or the implantation of telemetry devices which may compound and confound stress measures, however the technology for less invasive devices is developing rapidly. It is important to ensure that the taking of samples simply to measure welfare should not be contributing to the negative welfare state of the animal. Telomere attrition is a cellular biomarker of biological age which provides a molecular measure of cumulative experience that has been proposed to be used to assess the welfare impact of husbandry regimes on animals. The majority of studies on telomere length have been done on blood samples, but in some studies buccal cells have been used, which requires less invasive sampling [28]. As technologies develop, further markers may be assessed which do not in themselves have a negative impact on the animal simply for the sake of measuring it [29].

Another traditional measurement of physiological stress has focused on the detection of hypothalamic–pituitary–adrenal axis (HPA) hormones including cortisol in blood, faeces, urine, saliva, tears or hair. One difficulty in the measurement of cortisol can be the interpretation of results. The collection of faeces, urine or saliva samples for cortisol assay is practically complex, particularly from group-housed animals on a forage substrate and the interpretation of results is complicated by considerable individual variation [30], a natural circadian variation in cortisol levels [31,32,33], the fact that a cortisol response is associated with some non-stress stimuli, and the fact that some stress responses may not involve elevated cortisol levels [34]. These problems have contributed to an increasing dissatisfaction with the use of cortisol to measure stress levels [34,35].

Measurements such as the neutrophil activation assay or measurement of leukocyte cell counts can give an indication of the effect on the immune response. The measurement of acute phase proteins can also be applied to the assessment of welfare [36]. The leukocyte activation test measures the degree to which blood can produce a further neutrophil response (superoxide production) to an in vitro challenge. Animals under stress will produce a significantly lower leukocyte response than animals that are not stressed and this has been used to assess physiological stress levels in non-human primates [37]. However, this also requires the taking of blood samples.

Body weight (which must take into account age, sex, development/reproductive condition), and body condition scoring can both be used to assess the physical condition of the animal which will affect its welfare.

## 4. Behavioural Assessments

Behavioural measurements are critical in assessing the welfare of zoo animals. The first stage in assessing an animal’s wellbeing is to become familiar with the normal appearance and behavioural repertoire for that species. Animals have not evolved to live in man-made enclosures and the behaviour observed due to the constraints of the captive environment may not be the normal behavioural repertoire [38]. Behavioural observations can be made with different recording methods [39] but the goal is to ensure that animals have a natural frequency and range of behaviours and to take action to expand the behavioural repertoire by providing adequate environmental enrichment. Quantified behavioural measurement with systematic sampling allows monitoring of change. Some of the behavioural measures that can be used include quality of sleep [40], behaviours indicating boredom [41] and willingness to play [16,20,42].

Stress is a physiological and behavioural adjustment which aims to maintain homeostasis. It is possible to reduce and minimise stress levels but it has both benefits and harms to the individual, there is a point at which it will develop into distress. The question is always: how far? Where is the cut-off point? It will develop into distress when there is compromise on other biological processes such as growth, reproduction etc. Uncertainty may induce a state of distress, and such a situation may induce abnormal behaviours. The best known pathological forms of behaviour are behavioural stereotypies which are frequently observed in various species kept in captivity. Stereotypic behaviour is a form of behaviour thought to be induced by chronic frustration [43], often in response to a barren environment. It may manifest itself as extremes of behaviour (such as pacing, or repeated circling, head turning or self-harming) or as early signs of behavioural disturbance with a significant shift in the range and frequency of behaviours and the development of unusual, non-functional behaviour. The incidence of abnormal behaviours and inappropriate time budgeting, such as overgrooming, and the presence of neophobia are all parameters that can be measured. Trichotillomania is an example of an obsessive compulsive disorder associated with psychological stress, and in humans is relieved by anti-depressants. Many captive animals show forms of coat loss that are apparently absent in wild or free-living conspecifics, resulting from grooming or plucking behaviours directed at themselves or at other individuals. In primates, it is a pathological intensification of natural grooming behaviour, frequently with hair ingestion and is a symptom of psychogenic maladjustment to a poor environment, which can be reduced by the use of various types of environmental enrichment. Quantifying alopecia to assist with welfare assessment in primates (as in many species) can be done using a scoring system as shown in Honess et al. [44]. Mason and Latham [45] estimated that stereotypy prevalence for wild carnivores in zoos was 82% of individuals. However, their review concludes that stereotypic behaviour is not always associated with poor welfare and there may be beneficial consequences from performing the repetition to ameliorate welfare in a poor environment—a sort of “do it yourself enrichment”. Studies using voles kept in small cages showed that these stereotypies can be reduced through the use of the opioid antagonist naloxone [46]. This suggests that the occurrence of stereotypy is associated with the release of endorphins, which may elucidate the functional significance of stereotypies. However, even if stereotypies may be biologically significant to the animal, their incidence indicates that the animals have been (or still are) in a state of chronic stress. Hence, housing conditions within which stereotypies develop should be avoided.

There are various indicators which are useful in facilitating the goal of producing and assessing positive affective states in zoo animals including: controllability and predictability, individual temperament, social behaviours, goal directed behaviours, play behaviours, preference testing, consumer demand tests and cognitive bias tests, and anticipatory behaviour.

Thus, when considering stress, it is also necessary to review the animal’s controllability and predictability of relevant environmental changes. Chronic stress may occur when relevant environmental aspects have a low predictability and/or are not very well controllable over a long period of time. For example, animals may be able to predict the delivery of food, but the event may be beyond their control. The absence of control over an aspect of life as important as food can be a critical stress-eliciting factor in some husbandry systems. However, this does not mean that an ideal environment should be totally predictable and controllable and there is evidence that a certain degree of unpredictability is required to avoid the negative aspects of boredom.

Assessing what animals themselves choose and put value on in their environment can be a useful tool to assess welfare [47]. Kirkden and Pajor [48] proposed four important questions that need to be answered when investigating animal preference: whether the animal wants to avoid or obtain the resource; which resource it prefers among available resources; how strong this preference or absolute motivation is; and whether these preferences, or the strength of these preferences, are affected by environmental changes. Importantly, giving animals choices for short-term positive affective states must be balanced with assessment of long-term harm (e.g., ad libitum food access resulting in obesity) [13,49]. Giving animals access to what they value highest, either through preference testing or consumer demand studies [50,51] or, ideally, by providing a variety of resources that the animal can choose between depending on its affective state at the time, may improve zoo animal welfare.

With the modern age of readily accessible and affordable technologies, there is scope to provide much more control to zoo animals. This could include switches they operate to change temperatures, light levels and humidity; or even the scope to give animals tools which could let them better convey their needs to their human carers [49]. It is the duty of modern zoos to investigate these novel technologies and measure their effect on animal behaviour through robust behavioural monitoring strategies.

A challenge for zoo researchers is to collect enough data on the range of environmental parameters that are important for the huge variety of species kept in zoos, ideally from behavioural research performed in their wild environments, to inform management best practice. This would enable a large database of behavioural repertoires and natural history knowledge to be summated across varied taxonomic groups, to better inform our assessment of positive affective states. Determining indicators of good welfare for species in captivity is a challenge, as historically welfare has been assessed using wild conspecific behaviour as the baseline. However, it is important to recognise that captive wild animals in zoos generally lead very different lives to their wild counterparts, and their welfare needs in the captive situation will therefore need to be assessed to some extent independently of this wild data. Reviews have supported the idea that captive wild animals that are fully able to engage in their environments and to have positive social interactions through living in conditions that reflect their natural habitats may achieve high welfare and positive affective states, provided their basic physiological and psychological needs are appropriately met [19,20,47]. However, environments that fully mimic “the wild” are not necessarily better for welfare [51] and providing for optimal captive zoo animal welfare should provide some aspects of the wild environment (such as opportunity for foraging, exploration and choice) and withhold some of the stressors (such as presence of predators).

When developing factors to be scored for each species, there is huge variability in the available information about the natural biology for some species and even less information concerning those animals in captivity. Scimitar-horned oryx (*Oryx dammah*) have been regarded as an endangered species from the late 1980s before becoming extinct in the wild in the year 2000. Their natural habitat prior to extinction includes locations such as the Sahara and areas of vast desert and sub-desert (IUCN SSC Antelope Specialist Group, 2016) [52]. Thus, there is less available literature and documentation of the species’ natural behaviours and group structures in the wild, when compared to other captive zoological species. This makes the identification of factors to include within species-specific welfare assessment more challenging.

If captive animals exhibit different behaviours to the wild, should that always be an indicator of poor welfare [53]? Recognised wild behaviours and group sizes are sometimes used as baseline indicators of the very best welfare, but wild baselines are not always the most accurate indicator of what is right for an animal in captivity. For example, male Amur tigers in the wild would be solitary animals that only interact with conspecifics for mating and territory/resource disputes, whereas, in captivity, they seem to have improved welfare when they are part of a group [54]. “As wild” should not always be the starting point for the best welfare but further research needs to be undertaken before a clear baseline can be determined. Even if this were achieved, there would always be individuals for which “normal” is different and therefore defining factors for welfare scoring will always have its challenges.

When considering zoo animal welfare, each animal’s individual temperament or “personality” may play a role in its ability to cope with the captive environment. Not only does the reaction to a stressor vary, but also the type of reaction will differ among individuals. Many animal species, including humans, show different coping styles in response to a particular stressor. The distinction between types of individuals (such as proactive or reactive copers) plays a role in the development of a social hierarchy and the stability of social groups. This has been more widely researched in farm animals: one study in dairy ewes identified three emotional states: “fear susceptible”, “intermediate” and “calm, non-emotive ewes”, based on their responses to learning tasks and fear tests [55]. These ewes’ temperaments also determined their reproductive and production success and different reactions to maternal care of offspring, with the non-emotive ewes showing consistent care of their lambs while the emotive ewes showing increased anxiety states after giving birth [55]. Further studies have also supported that calm ewes give better maternal care [56]. Robinson et al. [57] found that captive chimpanzees showing indicators of positive welfare (as judged from survey results) tended to show more extraversion and lower neuroticism behaviours.

Bond-affirmation behaviours such as allo-grooming and other social behaviours such as sexual activity and maternal or group offspring care are also suggested to be associated with positive affective states [20,21,50]. Facial expressions and vocalisations such as purring or chirping may also suggest positive affective states and could be useful welfare indicators [13,21,40,58]. Zoos face the additional challenge of observing and understanding these behaviours in the captive environment, particularly as some of them may be too subtle for our human perception to detect, or outside our audible range.

Interactions with humans can contribute to negative or positive welfare [19,40,59,60]. Zoo animal training, conducted primarily through positive reinforcement training, can help to produce positive affects in zoo animals and potentially to improve staff attitudes to animals under their care [59,61]. Using positive reinforcement training and strengthening the animals’ trust in care staff can help to reduce the necessity of fear-driven management of zoo species (such as herding animals away from staff to lock them inside a house). Records of what training is undertaken and the responses and engagement of the animal should be included in welfare scoring assessments.

Promoting positive human-animal interactions in all aspects of zoo care has the potential to increase zoo animals’ welfare, and positive relationships may themselves offer enrichment to zoo animals [59]. Again, zoo staff need to recognise that what promotes positive welfare in domestic species can be very different in zoo species, particularly those that are wild-caught. Studies have shown that care staff interactions with zoo animals through barriers create a more favourable animal–staff relationship than interactions where care staff enter enclosures [59]. Further research into the visitor effects on zoo animal welfare are also needed [62]; while generally visitor presence or behaviours can act as stressors, the way in which visitors interact with zoo species can help to reduce this effect (such as crouching rather than standing in front of enclosures for primate species [63]). It would be prudent for zoos to investigate novel ways to promote positive welfare through public interactions, some examples of which have been documented [61,62].

Rich environments providing a variety of stimuli and opportunities for exploration, foraging, food acquisition and other goal-directed behaviours are likely to contribute to positive affective states in animals [20]. Inquisitive exploration and information gathering, which an animal performs to seek stimulation, is thought to be self-rewarding, and animals choose to perform these behaviours when their basic needs have been met [21]. Studies on contra-freeloading have shown that many animals in certain circumstances prefer to work for a resource such as food, rather than having “easy” access to it and that provision of problem-solving tasks can result in less negative affective states and abnormal behaviours [49].

Another measure identified as a potential indicator of positive welfare is play [16,20,21,42]. Bateson [64] suggested play is intrinsically motivated, occurring when the animal is healthy and not stressed, and that play behaviours in themselves are rewarding. He further categorised “playful play” (as opposed to the wider biological definition of play) as play occurring when the animal is in a positive affective state [64], and highlighted that it is often not possible for human observers to differentiate this type of play in animals. Play has been shown to be a reward for maze-learning in rats [65], while play behaviour in lambs was almost eliminated after castration [66]. Other studies [67] suggest it is currently not possible to determine whether play does indeed suggest improved welfare compared to neutral welfare states, and it is important to remember that not all species engage in behaviours that we interpret as play [42]. While generally negative affective states may reduce play activity, this is not always the case; there is evidence that play can also increase during some stressful situations and different types of play activity may occur under different affective states [42,67]. Therefore, currently, the link between play behaviours and positive affective state remains unclear [13], but data on its usefulness as a positive welfare indicator are increasing, including a number of studies looking at the effects of play in farmed pigs [50].

Assessing what is important to animals and recording their resulting behaviours, can be a useful tool in measuring affective states. These “tests” include preference testing, consumer demand tests and cognitive bias tests (or “judgement” tests) [50]. The capacity for zoos to use these tests as tools varies across species and is limited by aspects such as finances and available time for animal training. Offering animals’ choices and assessing their responses can help inform which resources zoo animals prefer in captive environments; this area has been studied in more depth in farmed animals such as mink [68]. A study of captive orang-utans found that they preferred to position themselves facing the window to view the visitor area [60]. Cognitive bias assessment may be a useful indicator of positive affective states in animals [69,70,71,72,73]. This is the idea, originally from human psychology research, that an animal with an “optimistic” bias (i.e., having a positive affective state) will react in tests more positively to neutral stimuli and vice versa [74]. Mendl et al. [70] reviewed this topic in domestic and non-domestic species, but while explored in domesticated animals [75,76], there have been limited studies in captive wildlife species.

While enriching environments can help animals to cope with future change and stressors, it is also possible that those animals which then move to more barren environments may suffer more than those that had never experienced enriched environments. Douglas et al. showed that pigs housed in enriched environments showed a more “optimistic” response to judgement bias tests; when they were then moved to a sparse environment they reacted more “pessimistically” in the tests compared to those pigs only housed in barren environments [77]. This is a particular concern for zoos, where animals are frequently moved between collections as part of breeding programmes or for management reasons, and the quality of enclosures at each location is not consistent. Captive rhesus macaques have shown differing responses to ambiguous stimuli compared to known stimuli during cognitive bias testing, depending on whether a positive (environmental enrichment) or negative (veterinary intervention) event preceded the test [78]—with macaques performing the test after a veterinary intervention reacting in a more pessimistic way to neutral stimuli while those that took the test after enrichment showed a more optimistic response. Similar studies in captive European starlings (*Sturnus vulgaris*) have supported this [79,80], and there are further examples in farm animals including lambs [81]. One study in squirrel monkeys found that lower levels of environmental enrichment resulted in more negative responses to a novel stimulus, but stated that the degree of variation of response between individuals was high, and enrichment should be tailored to the individual to be effective [82]. Other studies have focused on the expression of social behaviours and their correlation with optimistic performance in judgement bias tests, for example in dolphins, a highly social species [83]. Burman et al. [84] showed that in rats, even short-term decreases in anxiety states through environmental manipulation (in this case, decreased light intensity), can affect judgement bias, making rats behave more optimistically in tasks after only brief spells in lower-light environments, suggesting short-term effects are also important.

Cognitive bias testing in animals is a relatively new tool that researchers are using to investigate animal behaviour and welfare, and care is needed in the interpretation of these studies due to confounders and limited validation [74]. These tests require substantial training before the animals can perform them, and this in itself may modulate the animal’s affective state [74]. Cognitive bias assessment may prove challenging to perform in zoo species on a day-to-day basis, but it suggests that encouraging positive affective states through means such as social and environmental enrichment, as appropriate to the individual and the species, should result in animals that have a more positive reaction to novel stimuli and situations.

Anticipatory behaviour, a goal directed behaviour that occurs prior to the acquisition of a reward, may reflect how regularly zoo animals have positive experiences [85]. It may also help to produce a temporary positive affective state, which may be of importance to animals in fairly consistent environments [21,86,87]. A group studying grizzly bears found the bears that showed increased pacing prior to cognitive judgement testing showed more optimistic choices in these tests [88]. However, another study in dolphins found increased anticipatory behaviour for food-reward-based training sessions in individuals that showed more pessimistic responses in cognitive bias tests [89], and another showed that rats in more enriched environments showed less anticipatory behaviour to rewards than those living in standard environments [90]. These findings should be interpreted with caution due to differences between the tests. There is a definite need for zoos to examine all types of anticipatory behaviour in more detail, so that we can better understand the welfare implications of these behaviours.

## 5. Current Frameworks for Welfare Assessment in Zoos

Several examples of frameworks for zoo animal welfare assessment have recently been published in peer reviewed journals. These include frameworks focussed on organisational structure and staff roles [91], assessments for auditing individual species [92,93,94,95] and for monitoring class level taxonomic groups [96].

Kagan et al. [91] proposed a universal framework developed by the Detroit Zoological Society. This focuses on ensuring that techniques are developed within zoos to assess all potential indicators of welfare, including affective states. The importance of this has been highlighted by several others [11,97,98,99]. The advantage of this approach is that it starts with institutional philosophy and policy thereby ensuring that welfare programmes and developments are fully supported and appropriately resourced. The framework also highlights several important features of programme management, including taking an “animal-centred” approach where high quality of life experience for the individual is the over-riding factor in collection planning.

The emphasis on consideration of the entire 24 h life experience of zoo animals is shared by other proposed frameworks, in particular the “24/7” approach to zoo animal welfare proposed by Brando et al. [92]. This model for welfare assessment is an adaptation of the twelve welfare assessment criteria outlined in the Welfare Quality framework for food production animals [100,101,102]. They propose two new criteria relating to feeding and perceived control resulting in a tool with fourteen criteria for the assessment of each species. Natural history, biology, ecology, diet, habitat, social structure and activity patterns are some of the topics proposed to be taken into account when developing a species specific animal welfare programme. This evidence base may then be used to highlight potential mismatches between the wild and captivity. Consideration must be given to the 24 h experience of animals but practically monitoring this can be challenging to achieve.

Similarly, Koene [94] focused on using behavioural adaptations to assess zoo animal welfare. This approach involved developing databases of species characteristics and comparing behaviours in natural and captive environments to highlight welfare problems. The authors also suggest an adaptation of the Welfare Quality framework for use in giraffe and propose that a gold standard using nature as a reference may be possible, although others have suggested limitations using this approach [5].

The Welfare Quality framework was also adapted by Clegg et al. [93] to develop the “C-Well” assessment for use in captive bottle-nose dolphins (*Tursiops truncatus*). In this case, the assessment included eleven criteria and 36 species-specific measures. The authors highlight the importance of the inclusion of animal-based measures. Others have suggested the importance of animal-based measures, though cautioned that some physiological measures are not always practical to use in zoo animals [53]. This framework suggests that measures should be considered in light of the species under assessment, rather than trying to find a set of criteria which works for all.

Additionally, von Fersen et al. [95] proposed a decision tree for inspectors to use when assessing zoo animal welfare. This four-step approach begins with a survey including life history and current management. These data are then analysed and scored producing a preliminary report. The third stage involves an in-situ inspection where the survey data is verified and behavioural observations are undertaken alongside hormone analysis. The findings from this stage are then scored and conclusions drawn in the final stage. The framework was proposed as a result of discussions focussing on marine mammal welfare and was implemented for the assessment of captive bottle-nose dolphins and Antillean manatee (*Trichechus manatus manatus*).

The animal’s quality of life should be used for decision-making and to do this an objective assessment of quality of life is needed [103]. Combining a range of assessment parameters into one usable entity has been identified as an important goal in providing a practical, objective and robust assessment of welfare. The Animal Welfare Assessment Grid (AWAG) was developed for monitoring the welfare and cumulative lifetime experience of primates in research institutions [104,105]. The AWAG records physical health, psychological wellbeing, environmental comfort, and veterinary and management procedural events, encompassing the five domains of animal welfare, and drawing attention to the temporal component of welfare that is often overlooked which allows an assessment of quality of life affected by all the events that occur. The AWAG enables a numeric, as well as visual, representation of the animal’s welfare and represents a valuable tool for those tasked with oversight of, or monitoring of, animals in captivity. The structure of the AWAG makes it highly adaptable for any species. It was validated in experimental primates [105] and Justice et al. [96] successfully adapted the AWAG for use in zoos as a monitoring tool which highlights perceived positive and negative welfare impacts. These can then be investigated further using other auditing methods [96]. In a zoo setting, scores are generated using zoo animal care staff daily reports. This tool is computer based and possesses advantages over the farm animal Welfare Quality protocol [95] since it generates visual representations of welfare [92] which are potentially very useful to zoo managers for demonstrating current levels of welfare and informing management decisions [96].

In addition to these published studies, the AWAG has been trialled further within a zoological collection on giraffe (*Giraffa camelopardalis*), scimitar horned oryx (*Oryx dammah*) and large felids (namely Amur tiger (*Panthera tigris altaica*), Amur leopard (*Panthera pardus orientalis*), snow leopard (*Panthera uncial*) and cheetah (*Acinonyx jubatus*)), The first stage of these additional studies was identifying a set of species-specific factors to score within each of the four parameters defined by the AWAG tool. These factors were developed through discussions with experienced animal care staff, zoo veterinarians, and a zoologist at the collection, as well as extensive research into the species being studied to identify the most appropriate indicators of these species’ welfare in captivity. Research into known abnormal behaviours for each species was also carried out to ensure that the factors scored would pick up known species-specific issues. It is important to develop factors that will realistically and objectively assess the animal’s quality of life but which are also feasible and practical to measure within the constraints of the zoo environment. With the AWAG system, these factors can be easily adapted and improved for a species or individual animal over time as the tool is further validated.

The AWAG system can be used to look at either a group of animals or individuals and has been validated against both in these studies. Whether to assess the individual or the group will depend on how the information is captured by the animal care teams, how easy it is to identify an individual, and whether there are any specific concerns about an individual animal. The focus of the animal care staff should always be to look after the welfare and needs of the animals in their care and they only have a limited working day in which to do this. The continued planned development of the AWAG into a simple app based system would make it much easier for the care staff to enter a welfare score directly. The continued development of automated recording technologies to assess behavioural indicators which can link into the AWAG system will also assist with 24/7 monitoring.

The AWAG offers an evidence-based tool for continual welfare assessment, but it should be constantly adapted to include measures of good welfare, using technology where appropriate. For example, computer image recognition software is becoming highly detailed and has been used to assess facial grimace (as a proxy for pain/poor welfare) in mice [106]. This technology could therefore also be used to monitor positive facial expressions, once these have been determined, as an assessment of positive affective states [40]. Other technologies, such as auditory monitoring devices, closed circuit television (CCTV) recording and remote or invasive devices to measure heart rate, temperature and heart rate variability could also be used to detect both positive and negative states, and may soon be commercially available to zoo communities. Immune markers and monitoring the sleep patterns of animals could also provide information on the affective state [13,40]. While not practical in the zoo animal setting, research-based imaging techniques such as electroencephalography and functional magnetic resonance imaging studies, which have been used to investigate positive affective state and pleasure-centre responses in humans, could also be used in animals [13], but not if the effect of the monitoring decreases their welfare.

## 6. Conclusions

The majority of published studies on zoo animal welfare have focused on mammalian species [17,107]; however, it is necessary to perform evidence-based assessments of zoo welfare across taxonomic groups to inform the management strategy. The perception of zoo animal welfare should be from the animals’ perspective rather than our anthropogenic view, when aiming to ensure positive affective states. Much zoo husbandry and housing provision is based on what has worked previously (or is working currently) and this “status quo” is then adopted into best-practice guidelines, instead of from an evidence-based approach [17]. The challenge is to validate the measures of positive affect in zoo species, confounded further by the challenges of small sample sizes and challenging working environments for the collection of robust, repeatable experimental data [107]. Due to the paucity of wild data, it can be difficult, or even misleading, to use this to assess the welfare of zoo animals. However, the AWAG can be used to create a benchmark against which zoo managers can assess welfare improvements over time, which will assist with solving the issues around trying to create a “gold standard”. The AWAG is a highly adaptable tool which aims to assess each animal, as an individual, over the course of its life. Building the themes of positive affective state measurement into this welfare assessment ensures we are able to identify more than just compromised welfare, and thus improve our provision of conditions for positive welfare and life experience in zoo animals.

Attending to the welfare needs of animals is not a passive process; it requires a continuous planning, implementation, assessment, and revision cycle. The assessment of welfare facilitates not only the retrospective illustration of quality of life and the impact of enrichment, husbandry and procedures, but also helps enable the projection of future harms to the animal related to anticipated change, or lack of change. However the assessment of welfare alone is not sufficient and does nothing for the animal whose perception of its own welfare is not affected by the reason it is maintained (whether it is for exhibition or breeding use, for example) [108]. The outcome of monitoring and assessment must be action to improve welfare and the welfare assessment is simply the tool to demonstrate the action is effective. The continuing development of IT systems of activity monitoring which automatically link to animal unit databases will provide data in the future that can be used to quantify welfare and can be reviewed and reassessed at regular time points.

Zoos have competing priorities: to entertain; to engage and inspire the public to love the natural world and support conservation; to ensure revenue is created to pay for running costs, reinvestment and conservation project support; and to provide the animals in their care with a life worth living and ideally a good life [103]. Public perception of what contributes to good welfare in zoos is often conflicting and comes from the anthropocentric assessment of perceived welfare and enclosure aesthetics [109]. It is our duty to increase knowledge and understanding of animal behaviour, welfare, enclosure design and enrichment to improve the animals’ quality of life.
